# First record of *Licnophora chattoni* (Ciliophora, Spirotrichea) on the sabellid worm *Bispira melanostigma* from marina fouling communities

**DOI:** 10.3897/BDJ.14.e190347

**Published:** 2026-05-11

**Authors:** Jesús Angel de León-González, Rosaura Mayén-Estrada, Luis Fernando Carrera-Parra, María Elena García Garza, María Ana Tovar-Hernández

**Affiliations:** 1 Universidad Autónoma de Nuevo León, Facultad de Ciencias Biológicas, Laboratorio de Biosistemática, San Nicolás de los Garza, Nuevo León, Mexico Universidad Autónoma de Nuevo León, Facultad de Ciencias Biológicas, Laboratorio de Biosistemática San Nicolás de los Garza, Nuevo León Mexico; 2 Universidad Nacional Autónoma de México, Facultad de Ciencias, Departamento de Biología Comparada, Laboratorio de Protozoología, Ciudad de México, Mexico Universidad Nacional Autónoma de México, Facultad de Ciencias, Departamento de Biología Comparada, Laboratorio de Protozoología Ciudad de México Mexico; 3 El Colegio de la Frontera Sur, Departamento de Sistemática y Ecología Acuática, Chetumal, Mexico El Colegio de la Frontera Sur, Departamento de Sistemática y Ecología Acuática Chetumal Mexico; 4 Universidad Autónoma de Nuevo León, Facultad de Ciencias Biológicas, Laboratorio de Biosistemática, San Nicolás de los Garza, Mexico Universidad Autónoma de Nuevo León, Facultad de Ciencias Biológicas, Laboratorio de Biosistemática San Nicolás de los Garza Mexico

**Keywords:** Sabellidae, Annelida, Licnophorida, epibionts–basibionts, southern Gulf of Mexico

## Abstract

**Background:**

Epibiosis between ciliates and feather duster worms (Sabellidae) remains poorly known and has been documented in only four species, belonging to the genera *Eudistylia*, *Laonome*, *Schizobanchia* and *Sabella*. *Licnophora* is a genus of ciliates whose species live as epibionts on freshwater or marine organisms, involving algae, macrophytes and a wide range of marine animals from different phyla, including amongst them polychaete worms.

**New information:**

The tubiculous polychaete *Bispira melanostigma* is here reported for the first time as a component of fouling communities on a dock piling in the southern Gulf of Mexico, extending its known ecological occurrence beyond corals reefs, sand and seagrasses beds of the Caribbean Sea. In addition, ciliated epibionts attached to the radioles are reported and identified as *Licnophora chattoni*, constituting the first documented epibiotic association between this licnophorid ciliate and a sabellid fan worm.

## Introduction

Epibiosis is a facultative association between two organisms: the epibiont, which colonises the surface of a living substrate and the basibiont, which hosts the epibionts and provides living support (Wahl 1989, Wahl 2010, Ansari and Bhadury 2016, Mayén-Estrada et al. 2022). The first comprehensive review of epibiosis involving polychaete worms and peritrich ciliate protists was conducted by Mikac et al. (2019), who found 40 taxa of peritrich ciliates as epibionts of 48 polychaete taxa belonging to 18 families (Ampharetidae, Arenicolidae, Capitellidae, Flabelligeridae, Nephtyidae, Nereididae, Nerillidae, Onuphidae, Orbiniidae, Pectinariidae, Phyllodocidae, Polynoidae, Serpulidae, Sigalionidae, Spionidae, Syllidae, Terebellidae and Trichobranchidae).

Records of ciliate epibionts are often overlooked in studies on polychaetes and, consequently, available inventories require continuous updating. This is the case of the family Sabellidae (fan worms or feather duster worms), in which epibiotic ciliates are typically associated with the radioles of the branchial crowns and have been documented in only four host species: *Ignotocoma sabellarum* Kozloff, 1961 on *Schizobranchia insignis* Bush, 1905 (Bush 1905) and *Eudistylia polymorpha* (Johnson, 1901) (Johnson 1901, Kozloff 1961); *Colligocineta furax* Kozloff, 1965 on *Laonome kroyeri* Malmgren, 1866 (Malmgren 1866, Kozloff 1965); *Phalacrocleptes verruciformis* Kozloff, 1966 on *S. insignis* (Kozloff 1966); and *Colligocineta affinis* Kozloff, 1976 and *C. finleyi* Kozloff, 1976 on *Sabella pavonina* Savigny, 1822 (Savigny 1822, Kozloff 1976).

In addition, Bharatidasan et al. (2021) reported associations between ciliate protist and polychetes involving members of the families Nereididae, Terebellidae and Sabellariidae. Additionally, recently, Chatterjee et al. (2025) reported two species of *Cothurnia* Ehrenberg, 1831 (Ehrenberg 1831) as epibionts on unidentified species of polychaetes at 5004 m depth of the Indian Ocean, thus reaching the sum of 20 families of polychaetes after the contribution by Mikac et al. (2019).

*Bispira* Krøyer, 1856 (Krøyer 1856) is a genus of fan worms composed of solitary or gregarious species, some of which are conspicuous and visually attractive components of coral reef communities. The genus currently includes 27 species (Capa et al. 2021, Enrichetti et al. 2022, Tovar-Hernández et al. 2025). The social feather duster worm *Bispira melanostigma* (Schmarda, 1861) (Schmarda 1861) is a Caribbean species (Knight-Jones and Perkins 1998, Tovar-Hernández and Salazar-Vallejo 2006, Giangrande et al. 2007, Dean 2016), with a couple of records in the Pacific coasts of Costa Rica and Panama (Capa and López 2004, Dean et al. 2012) and the Gulf of California (Salazar-Vallejo and Stock 1987). In the present study, we report *B. melanostigma* for the first time as part of the fouling community of a marina, extending its known ecological occurrence to artificial substrates. In addition, we document the first record of this sabellid species acting as a basibiont for the ciliated protists *Licnophora chattoni* Villeneuve-Brachon, 1939 (Villeneuve-Brachon 1939) attached to the branchial crown, representing the first documented epibiotic association between a licnophorid ciliate and a sabellid fan worm.

## Materials and methods

Sampling was carried out in wood dock pilings of the Marina Terminal San Francisco de Campeche, Campeche (southern Gulf of Mexico) in 2021, under the permission issued by the Comisión Nacional de Acuacultura y Pesca (DGOPA.14011.151012.3291). Collection, relaxation and fixation (ethanol 96%) procedures followed the standardised protocol by Pech et al. (2023). Sabellids were identified to species level, based primarily on the keys provided by Knight-Jones and Perkins (1998), Tovar-Hernández and Salazar-Vallejo (2006) and Tovar-Hernández and Fitzhugh (2021). Observations of sabellids were done using a Leica MZ75 stereomicroscope and an Olympus CH30 high power microscope. Photographs were taken with a Canon EOS Rebel T7i digital camera attached to the microscopes.

Sabellid radioles were separated to observe ciliates with a DIC Labophot2 Nikon microscope and microphotographs were obtained with a Rising Tech camera adapted to the microscope. Ciliated protists were stained with haematoxylin and trichrome techniques (Lee et al. 1985) for a detailed cellular observation. We followed the taxonomy proposed by Lynn (2008) and the identification was based on da Silva-Neto et al. (2012).

For scanning electron microscopy (SEM) analysis, radioles were dehydrated through a graded ethanol series, followed by replacement with increasing concentrations of hexamethyldisilazane (HMDS). After air-drying, they were mounted on aluminium stubs, sputter-coated with gold and examined using a JEOL JSM-6010Plus-LA SEM at the Scanning Electron Microscopy Laboratory (LMEB), ECOSUR, Chetumal.

Voucher specimens of sabellids and peritrichs were deposited in the Colección Poliquetológica de la Universidad Autónoma de Nuevo León (NL-INV-0002-05-09) and Colección de Bentos Costero (ECOSUR), El Colegio de la Frontera Sur, unidad Chetumal.

## Taxon treatments

### Bispira melanostigma

(Schmarda, 1861)

1D7F2ED3-810E-5084-A851-111989D08246

Sabella melanostigma Schmarda, 1861: 36, pl. 22, fig. 190.— Uebelacker (1984): 54–42, 54–43.— Salazar-Vallejo and Stock (1987): 271.
Sabella variegata Krøyer, 1856: 29–30 *fide* Knight-Jones and Perkins (1998): 415.
Sabella thoracica Krøyer, 1856: 31–32 *fide* Knight-Jones and Perkins (1998): 415.
Sabella bipunctata Baird, 1865 (Baird 1865): 158–159 *fide* Knight-Jones and Perkins (1998): 415.
Bispira melanostigma .— Knight-Jones and Perkins 1998: 415–419, figs. 11–12.— Capa and López (2004): 69–70.— Tovar-Hernández and Salazar-Vallejo (2006): 33–35, fig. 5.— Giangrande et al. (2007): 45, fig. 1B.— Dean et al. (2012): 364.— Dean (2016): 141.

#### Materials

- **Type status:** Other material. **Occurrence:** catalogNumber: UANL-8282; occurrenceRemarks: Found attached to wood piling; recordedBy: Anabel León Hernández, José Ángel García Trasviña; individualCount: 1; occurrenceID: A2838581-4C3C-596C-A5A5-37D2FFDC5293; **Taxon:** phylum: Annelida; class: Polychaeta; order: Sabellida; family: Sabellidae; genus: Bispira; specificEpithet: melanostigma; vernacularName: social feather duster worm; taxonomicStatus: accepted; **Location:** higherGeography: North America; Gulf of Mexico; Mexico; Campeche; Marina Terminal San Francisco Campeche; continent: America; waterBody: Gulf of Mexico; country: Mexico; countryCode: MX; stateProvince: Campeche; locality: Marina Terminal San Francisco de Campeche; minimumDepthInMeters: 0.57 m; decimalLatitude: 19.863083; decimalLongitude: -90.530936; **Identification:** identifiedBy: Tovar-Hernández; **Event:** eventDate: 13-03-2021; year: 2021; month: 03; day: 13; habitat: Submerged wood piling; fieldNumber: CAM-PS-A2-S/ 20210313-1; **Record Level:** institutionID: UANL; collectionID: NL-INV-0002-05-09
- **Type status:** Other material. **Occurrence:** catalogNumber: UANL-8283; occurrenceRemarks: Found attached to wood piling; recordedBy: Anabel León Hernández, José Ángel García Trasviña; individualCount: 4; occurrenceID: 700686C0-7328-5458-89F2-A98FA25A5683; **Taxon:** phylum: Annelida; class: Polychaeta; order: Sabellida; family: Sabellidae; genus: Bispira; specificEpithet: melanostigma; vernacularName: social feather duster worm; taxonomicStatus: accepted; **Location:** higherGeography: North America; Gulf of Mexico; Mexico; Campeche; Marina Terminal San Francisco Campeche; continent: America; waterBody: Gulf of Mexico; country: Mexico; countryCode: MX; stateProvince: Campeche; locality: Marina Terminal San Francisco de Campeche; minimumDepthInMeters: 0.57 m; decimalLatitude: 19.863083; decimalLongitude: -90.530936; **Identification:** identifiedBy: Tovar-Hernández; **Event:** eventDate: 13-03-2021; year: 2021; month: 03; day: 13; fieldNumber: CAM-PS-A2-S-20210313-1; **Record Level:** institutionID: UANL; collectionID: NL-INV-0002-05-09
- **Type status:** Other material. **Occurrence:** catalogNumber: UANL-8284; occurrenceRemarks: Found attached to wood piling; recordedBy: Anabel León Hernández, José Ángel García Trasviña; individualCount: 1; occurrenceID: 2B101106-8812-534B-B5BE-F6FF2EA29E37; **Taxon:** phylum: Annelida; class: Polychaeta; order: Sabellida; family: Sabellidae; genus: Bispira; specificEpithet: melanostigma; vernacularName: social feather duster worm; taxonomicStatus: accepted; **Location:** higherGeography: North America; Gulf of Mexico; Mexico; Campeche; Marina Terminal San Francisco Campeche; continent: America; waterBody: Gulf of Mexico; country: Mexico; countryCode: MX; stateProvince: Campeche; locality: Marina Terminal San Francisco de Campeche; minimumDepthInMeters: 0.57 m; decimalLatitude: 19.863083; decimalLongitude: -90.530936; **Identification:** identifiedBy: Tovar-Hernández; **Event:** eventDate: 13-03-2021; year: 2021; month: 03; day: 13; fieldNumber: CAM-PS-A3-Q/20210313-3; **Record Level:** institutionID: UANL; collectionID: NL-INV-0002-05-09

#### Description

**Figure 1.**
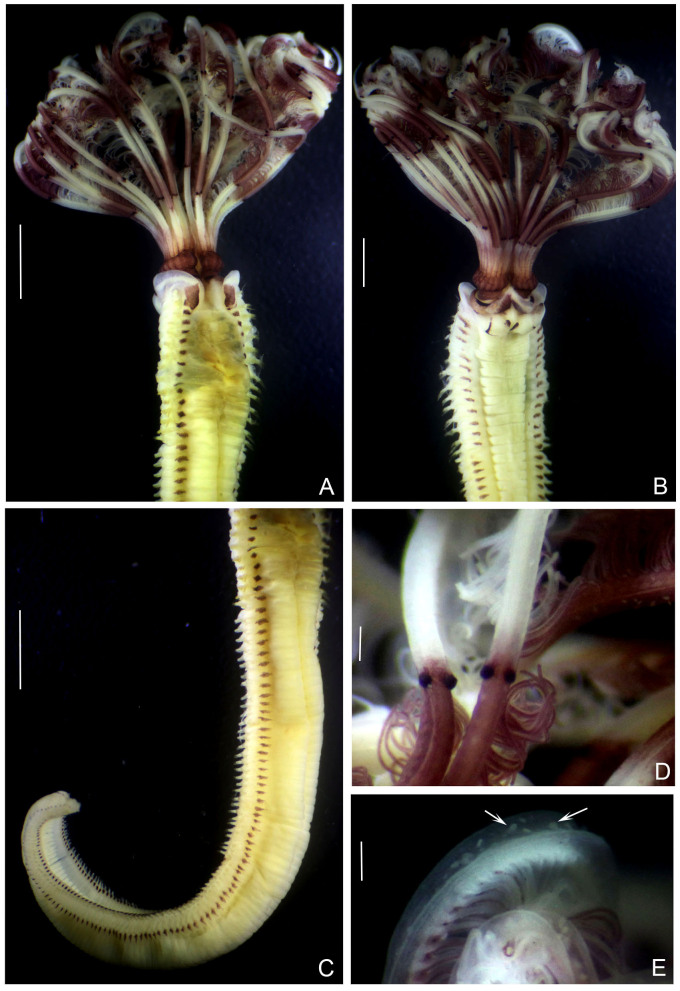
*Bispira melanostigma* (Schmarda, 1861) (UANL-8283). **A** Thorax and crown, dorsal view; **B** Same, ventral view; **C** Trunk, latero-dorsal view; **D** Radiolar eyes; **E** Radiolar tips with flanges forming a deep groove with ciliated protists as indicated with arrows. Scale bars: A, 2 mm; B, 1.5 mm; C, 2.5 mm; D, 0.2 mm; E, 0.2 mm.

Body with purple spots in dorsal collar margin (Fig. 1). Body length 22.32 mm excluding crown (13.6–34 mm), mid-thorax width 2.92 mm (2.2–4.5 mm). Radiolar crown with purple bands alternated with white bands (Fig. 1A and B). Radiolar crown length 9.06 mm (7.2–10.8 mm, n: 5). Twelve radiolar pairs (9–16 pairs) with ventral margin involuted ventrally in largest specimens. Radioles with 2–4 pairs of black, radiolar eyes (Fig. 1D). From mid-radiole length to the tips, flanges are broader than basal part, directed up forming a furrow or channel, in which ciliated protozoan are attached (Fig. 1E). Dorsal lips tapered. Dorsal collar margins prominent, dorsally separated by a broad gap (Fig. 1A). Lateral collar margins covering junction between thorax and crown (Fig. 1A), with a yellowish band parallel to dorsolateral margins. Ventral lappets rounded, with purple spots in its margins (Fig. 1B). Thoracic chaetigers 14 (13–15). Ventral shields separated from tori by broad gaps (Fig. 1B). Purple spots above each thoracic and abdominal fascicles (Fig. 1A and C) and between ventral shields and thoracic tori (Fig. 1B). Abdominal chaetigers 99 (81–117). Pygidium bilobed. All chaetae and uncini as described by Knight-Jones and Perkins (1998).

#### Ecology

**Figure 2.**
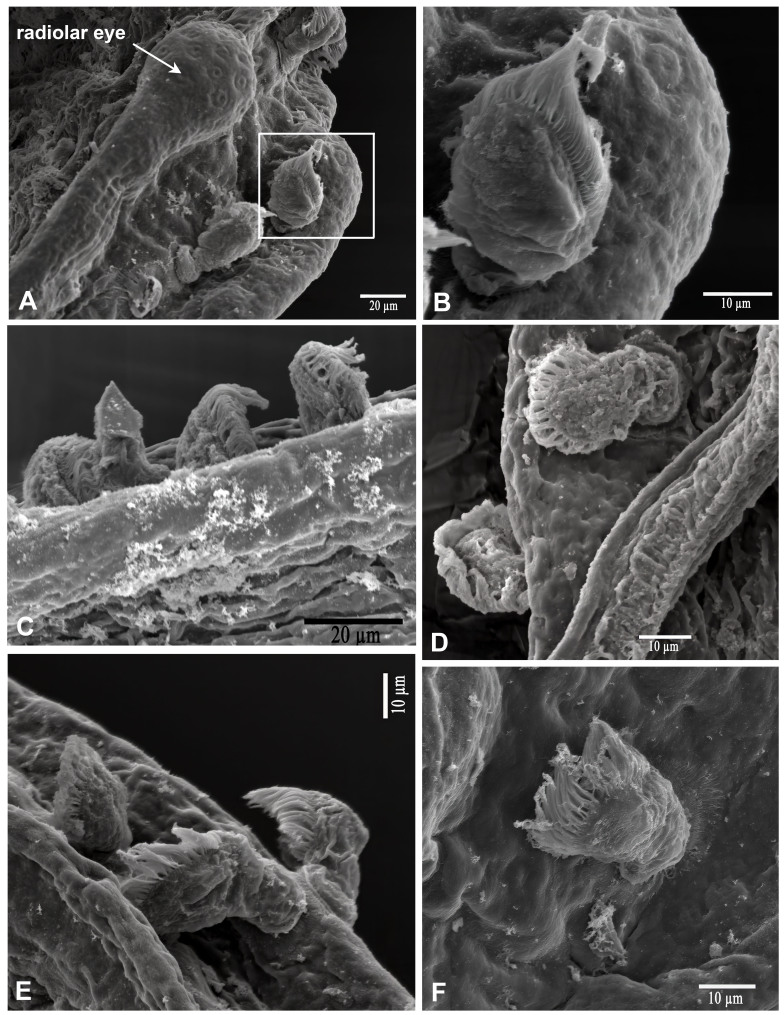
Scanning electron micrographs of *Licnophora chattoni* on the radioles of *Bispira melanostigma* (ECOSUR). **A–F** Different views of *Licnophora chattoni*.

Ciliated protists were found attached to the radiolar crowns in only two of the eight examined specimens, occurring primarily from the mid-length of the radioles to their distal tips (Fig. 2). These specimens were identified as *Licnophora chattoni* Villeneuve-Brachon, 1939, a member of the family Licnophoridae Bütschli, 1887 (Bütschli 1887), as stated below.

### Licnophora chattoni

Villeneuve-Brachon, 1939

707F6610-5A59-5390-903D-366864F8FD90

Licnophora chattoni Villeneuve-Brachon, 1939: 1362–1364, figs. 1–7, re-described by da Silva-Neto et al. (2012).

#### Materials

- **Type status:** Other material. **Occurrence:** catalogNumber: UANL-8283; recordedBy: Anabel León Hernández and José Ángel García Trasviña; individualID: 18; occurrenceID: 0B0FBEAB-49B3-5792-85AB-DE36387D8153; **Location:** higherGeographyID: North America, Gulf of Mexico; higherGeography: Gulf of Mexico, Campeche; continent: America; waterBody: Gulf of Mexico; country: Mexico; countryCode: MX; stateProvince: Campeche; locality: Marina Terminal San Francisco de Campeche; verbatimDepth: 0.57 m; decimalLatitude: 19.863083; decimalLongitude: -90.530936; **Event:** eventDate: 13-03-2021; year: 2021; month: 3; day: 13; habitat: Wood piling; fieldNumber: CAM-PS-A2-S-20210313-1; **Record Level:** institutionID: UANL; collectionID: NL-INV-0002-05-09

#### Description

**Figure 3.**
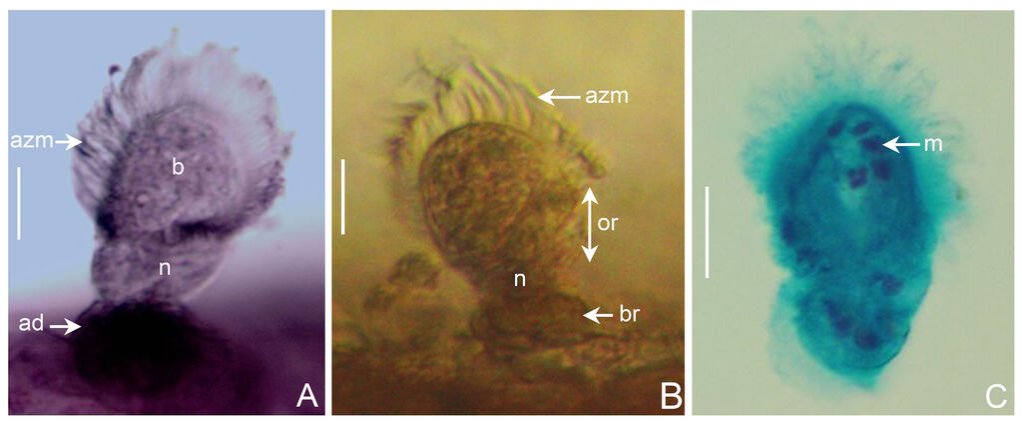
*Licnophora chattoni* Villeneuve-Brachon, 1939 (UANL-8283). **A** Cell regions: body (b), neck (n), adhesive disc (ad), adoral zone of membranelles (azm); **B** Oral region (or) with adoral zone of membranelles (azm), the neck (n) and basal region (br); **C** Segment of moniliform macronucleus (m) is shown. A - Ciliate stained with haematoxylin; B - individual fixed with ethanol 70%; C - cell stained by the trichrome technique. Scale bars 10 µm.

Cells consisting of three regions: body (b), neck (n) and aboral adhesive disc (ad) (Fig. 3A). Length of the cell (including the three regions) is between 40–58 µm and width of the body is 15–30 µm and, for the aboral adhesive disc, the diameter is between 10 and 41 µm, which is attached to the radioles through basal region (Fig. 3B, br). The peristome is very large and presents 55–125 membranelles forming the adoral zone of membranelles of oral polykinetids (Fig. 3A–B, azm), encircling oral region. Somatic cilia absent, except for posterior ciliary rings, encircling the attachment disc. Macronucleus irregularly moniliform with 8–13 segments arranged in a horseshoe-shape (Fig. 3C, m); micronucleus was not observed.

## Discussion

The genus *Licnophora* comprises 17 species, most of which have been found as epibionts on freshwater or marine organisms, including algae, macrophytes and a wide range of marine animals from different phyla, such as ascidians, bivalves, cnidarians, echinoderms, fishes, gastropods, hydrozoans, nudibranchs, platyhelminths, polychaetes and seahorses (Stevens 1904, da Silva-Neto et al. 2012).

Only three species of *Licnophora* have been reported in epibiosis with polychaetes. Claparède (1867) reported *L. cohnii* attached to the radioles of the serpulid *Protula tubularia* (Montagu, 1803) (Montagu 1803) (originally reported as *Psygmobranchus protensus* not Gmelin, sensu Philippi (1844)) (Philippi 1844). In addition, early records of *Licnophora auerbachii* Cohn, 1866 (*Cohn 1866*) associated with *Syllis* (Syllidae) were provided by Fabre-Domergue (1888) and Stevens (1904).

In the present study, *L. chattoni* is recorded for the first time from the southern Gulf of Mexico, associated with the branchial crown of the sabellid worm *B. melanostigma*, constituting the first documented epibiotic association between these two taxa. *Licnophora chattoni* was originally described by Villeneuve-Brachon (1939) from Banyuls sur mer (France), where it was found detached from the body walls of the ascidian *Phallusia mammillata* (Cuvier, 1815) (Cuvier 1815). Subsequently, da Silva-Neto et al. (2012) reported high densities of *L. chattoni* on the tentacles and hydranths of the tubulariid hydroid *Zyzyzzus warreni* Calder, 1988 (Calder 1988) (Cnidaria, Hydrozoa, Tubulariidae) from São Sebastião, Brazil, providing the most complete morphological re-description and illustrations of the species known until today. In total, three species of *Licnophora* are currently known to occur as epibionts of annelid polychaetes: *L. auerbachii* on *Syllis* (Syllidae; Fabre-Domergue (1888), Stevens (1904)), *L. chattoni* on *B. melanostigma* (Sabellidae; present study) and *L. cohnii* on *Protula tubularia* (Serpulidae; Claparède (1867)).

Members of Sabellidae, are sessile, tube-dwelling polychaetes that extend their radiolar crowns into the water column, exposed to currents, predators (Tilic et al. 2021), parasitic copepods (Capa et al. 2019) and epibiotic ciliates (Kozloff 1961, Kozloff 1965, Kozloff 1966, Kozloff 1976, present study). The crown is composed of two branchial lobes located laterally on either side of the mouth. Radioles are fused distally to these lobes and, from the inner margins of each radiole, paired series of ciliated pinnules branch out (Nicol 1931, Fitzhugh 1989). This configuration allows the capture of suspended food particles including plankton, debris and other suspended particles and their transportation aided by cilia towards the mouth. However, the function of the crown is not only for feeding, it is also used by the animal for respiration; to sort, capture and transfer particles with its ciliated inner margins for tube building (Nicol 1931, Bonar 1972); to brooding larvae in some taxa (Capa et al. 2019) or to collect and move sperm from the water column to epidermal spermathecae at the base of the crown in some species, where they are stored until later fertilisation of eggs (Rouse 1996). For most of the time, the crowns are exposed outside their tubes to the water column, with escape behaviour by retracting into their tubes. These offer a suitable microhabitat to other creatures, offering protection, shading, a resting place, a substrate for fixation or a source of food (capture or stealing of food particles when these are directed from the radioles to the worm mouth). Despite the multifunctional actions of the crowns, the comprehension of the relationships between ciliate epibionts and hosts is poorly understood, limiting their knowledge to occasional reports of species only.

Additionally, *B. melanostigma* is herein reported for the first time as a component of the marina fouling communities. This species was previously known from coral reefs, sandy substrates and seagrasses beds (Knight-Jones and Perkins 1998, Capa and López 2004, Tovar-Hernández and Salazar-Vallejo 2006, Giangrande et al. 2007, Dean 2016) and its occurrence on artificial structures expands its known ecological range.

## Supplementary Material

XML Treatment for Bispira melanostigma

XML Treatment for Licnophora chattoni

## References

[B13938829] Ansari K. G. M. T., Bhadury P. (2016). Occurrence of epibionts associated with meiofaunal basibionts from the world’s largest mangrove ecosystem, the Sundarbans. Marine Biodiversity.

[B14164341] Baird W. (1865). On new tubicolous annelids, in the collection of the British Museum. Part 2. Journal of the Linnean Society of London.

[B13938838] Bharatidasan V., Sarathy P. P., Murugesan P, de Matos Nogueira J. M. (2021). New records for associations between peritrich protozoan ciliates (Ciliophora, Sessilida) and polychaete worms (Annelida) from off the southeastern coast of India. Zootaxa.

[B14164316] Bonar D. B. (1972). Feeding and tube construction in *Chone
mollis* Bush (Polychaeta, Sabellidae). Journal of Experimental Marine Biology and Ecology.

[B14164350] Bush K. J. (1905). Tubicolous annelids of the tribes Sabellides and Serpulides from the Pacific Ocean. Harriman Alaska Expedition.

[B14166783] Bütschli O., Bronn H. G. (1887). Klassen und Ordnungen des Thier-Reichs..

[B14163007] Calder D. R. (1988). Shallow-water hydroids of Bermuda: the Athecatae. Royal Ontario Museum Life Sciences Contributions.

[B13938857] Capa M., López E. (2004). Sabellidae (Annelida: Polychaeta) living in blocks of dead coral in the Coiba National Park, Panamá. Journal of the Marine Biological Association of the United Kingdom.

[B14164276] Capa M., Giangrande A., Nogueira J. M. M., Tovar-Hernández M. A., Purschke G., Böggemann M., Westheide W. (2019). Handbook of Zoology, Annelida, Vol. 2: Pleistoannelida, Sedentaria II.

[B13938847] Capa María, Kupriyanova Elena, Nogueira João Miguel de Matos, Bick Andreas, Tovar-Hernández María Ana (2021). Fanworms: Yesterday, Today and Tomorrow. Diversity.

[B14163046] Chatterjee T., Sautya S., Gaikwad S., Mishra G. K., Abibulaeva A., Dovgal I. (2025). Report of ciliate epibiont (Ciliophora) on polychaetes (Polychaeta) at the depth of 5004 m from the Indian Ocean and notes on deep-sea epibiont ciliates of benthic small invertebrates. Cahiers de Biologie Marine.

[B13938866] Claparède E. (1867). Miscellanées Zoologiques. VI Sur les *Licnophora*, nouveau genre de la famille urcéolariens (infusoires ciliés). Annales des Sciences Naturelles, Zoologie et Paléontologie.

[B14162978] Cohn F. (1866). Neue Infusorien im Seeaquarium. Zeitschrift für wissenschaftliche Zoologie.

[B14163016] Cuvier G. (1815). Memoire sur les ascidies et sur leur anatomique. Mémoires du Muséum National d'Histoire Naturelle Paris.

[B13938875] da Silva-Neto Inácio Domingos, da Silva Paiva Thiago, Pedroso Dias Roberto Júnio, Alexandre Campos Carlos José, Migotto Alvaro Esteves (2012). Redescription of *Licnophora
chattoni* Villeneuve-Brachon, 1939 (Ciliophora, Spirotrichea), associated with *Zyzzyzus
warreni* Calder, 1988 (Cnidaria, Hydrozoa). European Journal of Protistology.

[B13938894] Dean Harlan K., Sibaja-Cordero Jeffrey A., Cortés Jorge (2012). Polychaetes (Annelida: Polychaeta) of Cocos Island National Park, Pacific Costa Rica. Pacific Science.

[B13938885] Dean Harlan K (2016). Some intertidal and shallow water polychaetes of the Caribbean coast of Costa Rica. Revista de Biología Tropical.

[B14164386] Ehrenberg C. G. (1831). Zur erkenntniss der Organisation in der Richtung des kleinsten Raumes. Zweiter Beitrag. Entwicklung, Lebensdauer und Structur der Magenthiere und Räderthiere oder sogenannten Infusorien, nebst einer physiologischen Characteristik beider Klassen und 412 Arten derselben. Vorgetragen in der Akademie der Wissenschaften zu Berlin im Jahre 1831..

[B13938903] Enrichetti Francesco, Baldrighi Elisa, Bavestrello Giorgio, Betti Federico, Canese Simonepietro, Costa Andrea, del Pasqua Michela, Giangrande Adriana, Langeneck Joachim, Misic Cristina, Putignano Matteo, Toma Margherita, Bo Marzia (2022). Ecological role and phylogenetic position of a new habitat-forming species (Canalipalpata, Sabellidae) from the Mediterranean mesophotic soft bottoms. Estuarine, Coastal and Shelf Science.

[B13938921] Fabre-Domergue P. (1888). Étude sur l’organisation des Urcéolaires et sur quelques genres d’infusoires voisins de cette famille. Journal de l’Anatomie et de la Physiologie Normales et Pathologiques de l’Homme et des Animaux.

[B14164298] Fitzhugh K. (1989). A systematic revision of the Sabellidae-Caobangiidae-Sabellongidae complex (Annelida: Polychaeta). Bulletin of the American Museum of Natural History.

[B13938930] Giangrande A, Licciano M, Gambi M C (2007). A collection of Sabellidae (Polychaeta) from Carrie Bow Cay (Belize, western Caribbean Sea) with the description of two new species. Zootaxa.

[B14164359] Johnson H. P. (1901). The Polychaeta of the Puget Sound region. Proceedings of the Boston Society for Natural History.

[B13938939] Knight-Jones P, Perkins T. H (1998). A revision of *Sabella*, *Bispira* and *Stylomma* (Polychaeta: Sabellidae). Zoological Journal of the Linnean Society.

[B13938948] Kozloff EUGENE N. (1961). A new genus and two new species of ancistrocomid ciliates (Holotricha: Thigmotricha) from sabellid polychaetes and from a Chiton. The Journal of Protozoology.

[B13938957] Kozloff EUGENE N. (1965). *Colligocineta
furax* gen. nov., sp. nov., an ancistrocomid ciliate (Holotricha: Thigmotricha) from the sabellid polychaete *Laonome kröyeri* Malmgren. The Journal of Protozoology.

[B13938966] Kozloff EUGENE N. (1966). *Phalacrocleptes
verruciformis* gen. nov., sp. nov., an unciliated ciliate from the sabellid polychaete *Schizobranchia
insignis* Bush. The Biological Bulletin.

[B13938975] Kozloff Eugene N. (1976). New ancistrocomid ciliates from polychaete annelids. Transactions of the American Microscopical Society.

[B13938984] Krøyer H. (1856). Bidrag til Kundskab af Sabellerne. Kongelige Danske Videnskabernes Selskabs Forhandlinger.

[B13938993] Lee J. J., Small E. B., Lynn D. H., Bovee E. C., Lee J. J., Hutner S. H., Bovee E. C. (1985). An illustrated guide to the protozoa.

[B13939006] Lynn D. H. (2008). The ciliated Protozoa. Characterization, classification, and guide to the literature.

[B14164368] Malmgren A. J. (1866). Nordiska Hafs-Annulater. [part three of three].. Öfversigt af Königlich Vetenskapsakademiens förhandlingar Stockholm.

[B13939023] Mayén-Estrada R, Pedroso Dias RJ, Ramírez-Ballesteros M, Rossi M, Reyes-Santos M, Durán-Ramírez CA, Cruz-Jiménez G, Pereira L, Mendes Gonçalves M. A (2022). Plankton Communities.

[B13939039] Mikac Barbara, Semprucci Federica, Guidi Loretta, Ponti Massimo, Abbiati Marco, Balsamo Maria, Dovgal Igor (2019). Newly discovered associations between peritrich ciliates (Ciliophora: Peritrichia) and scale polychaetes (Annelida: Polynoidae and Sigalionidae) with a review of polychaete–peritrich epibiosis. Zoological Journal of the Linnean Society.

[B14164325] Montagu G. (1803). Testacea Britannica or natural history of British shells, marine, land, and fresh-water, including the most minute: Systematically arranged and embellished with figures..

[B14164307] Nicol E. (1931). The feeding mechanism, formation of the tube, and physiology of digestion in *Sabella
pavonina*. Earth and Environmental Science Transactions of the Royal Society of Edinburgh.

[B14163077] Pech D, Tovar-Hernández M. A, Bastida-Zavala JR, Carrera-Parra L. F, Delgado-Blas VH, de León-González J. A, Díaz-Castañeda V, Galván-Villa C. M, García-Garza M. E, Martínez-Arce M, Salazar-Silva P, Salazar-Vallejo S. I (2023). Protocolo para el muestreo, análisis y estudio la biota portuaria en México.

[B14164333] Philippi A. (1844). Einige Bemerkungen über die Gattung Serpula, nebst Aufzählung der von mir im Mittelmeer mit dem Thier beobachteten Arten.

[B14164289] Rouse G. W. (1996). Variability of sperm storage by females in the Sabellidae and Serpulidae (Polychaeta, Sabellida. Zoomorphology.

[B13939068] Salazar-Vallejo S. I, Stock JH (1987). Apparent parasitism of *Sabella
melanostigma* (Polychaeta) by *Ammothella
spinifera* (Pycnogonida) from the Gulf of California. Revista de Biología Tropical.

[B14164377] Savigny J. -C. (1822). Système des annélides, principalement de celles des côtes de l'Égypte et de la Syrie, offrant les caractères tant distinctifs que naturels des Ordres, Familles et Genres, avec la Description des Espèces.. Description de l'Égypte ou Recueil des Observations et des Recherches qui ont été faites en Égypte pendant l'Expédition de l'Armée Française, publié par les Ordres de sa Majesté l'Empereur Napoléon le Grand, Histoire Naturelle..

[B13939078] Schmarda L. K. (1861). Neue Wirbellose Thiere Beobachtet und Gesammelt auf einer Reise um die Erde 1853 bis 1857. Neue Turbellarien, Rotatorien und Anneliden.

[B13939094] Stevens NM (1904). Further studies on the Ciliate *Infusoria*, *Lichnophora* and *Boveria*. Archiv für Protistenkunde.

[B14164267] Tilic E., Rouse G. W., Bartolomaeus T. (2021). Comparative ultrastructure of the radiolar crown in Sabellida (Annelida. Zoomorphology.

[B13939103] Tovar-Hernández MA, Fitzhugh K, de León-González JA (2021). Anélidos marinos de México y América Tropical..

[B13939116] Tovar-Hernández M. A., Salazar-Vallejo S. I. (2006). Sabellids (Polychaeta: Sabellidae) from the Grand Caribbean. Zoological Studies.

[B13939125] Tovar-Hernández M. A., De León-González J. A., Hendrickx M. E. (2025). Polychaeta collected during the research cruises TALUD aboard the R/V “El Puma” in the Mexican Pacific: Sabellidae and Serpulidae. Zootaxa.

[B13939134] Uebelacker JM, Uebelacker JM, Johnson PG (1984). Taxonomic guide to the polychaetes of the Northern Gulf of Mexico, Final Report to the Minerals Management Service, Contract 14––12–00129091..

[B13939156] Villeneuve-Brachon S. (1939). Sur la division et la formation du péristome des *Licnophora* (*L.
chattoni* n. sp.) (Ciliés hétérotriches). Comptes Rendus de l'Académie des Sciences, Paris.

[B13939165] Wahl M (1989). Marine epibiosis. I. Fouling and antifouling: some basic aspects. Marine Ecology Progress Series.

[B13939183] Wahl M, Dürr S, Thomason JC (2010). Biofouling.

